# Supplementary figures and images for: FPGA-based systolic deconvolution architecture for upsampling
Source: PeerJ Comput Sci. 2022 May 11;8:e973. doi: 10.7717/peerj-cs.973 (PMC9138038; doi:10.7717/peerj-cs.973)

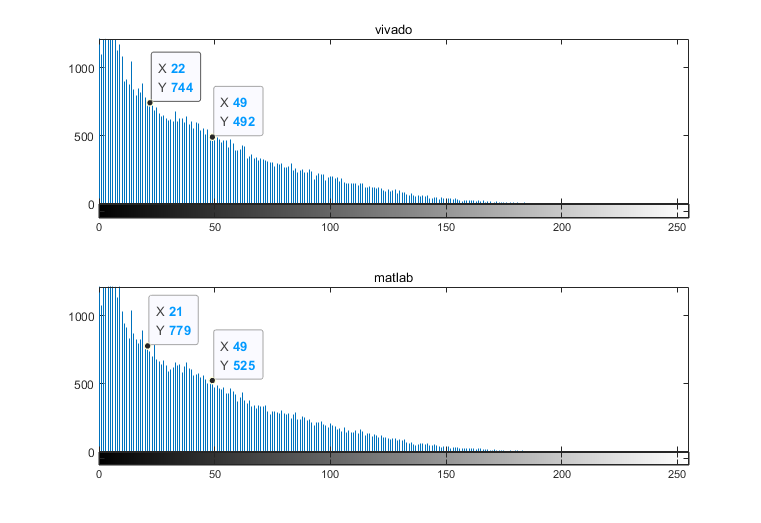

Supplement: Supplemental Information 3 — The histogram of the images obtained from Matlab and FPGA for realistic comparison [file peerj-cs-08-973-s003.zip › 3by3kernel/hist128-256-3.png]

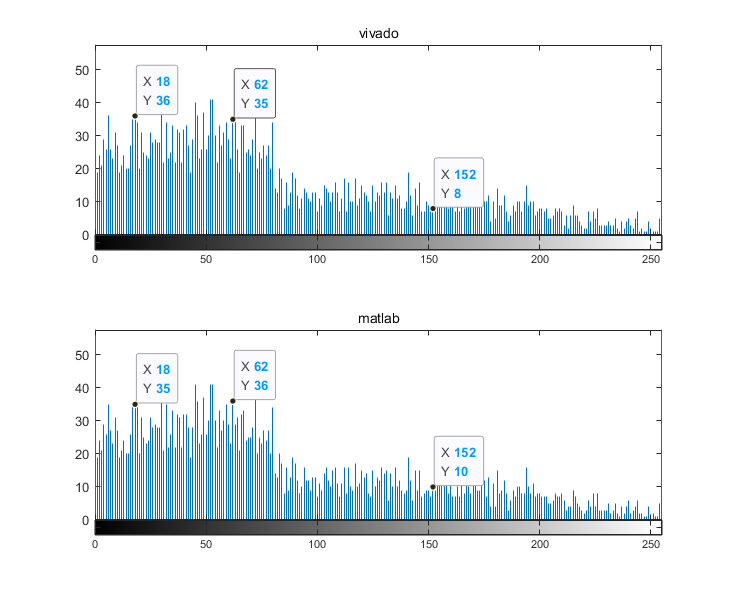

Supplement: Supplemental Information 3 — The histogram of the images obtained from Matlab and FPGA for realistic comparison [file peerj-cs-08-973-s003.zip › 3by3kernel/hist32-64-3.png]

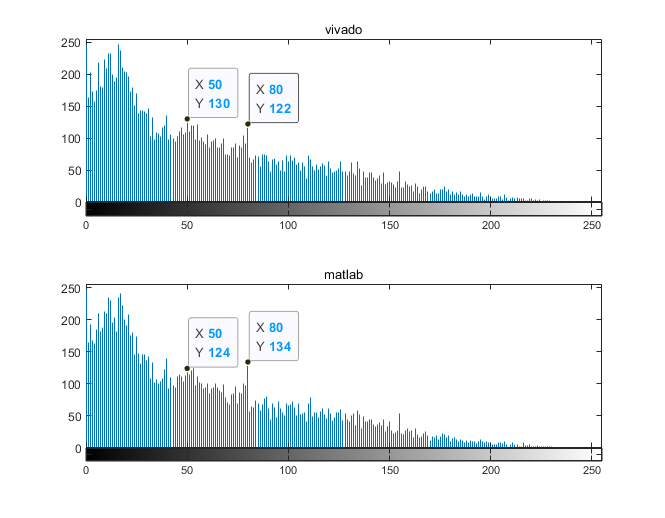

Supplement: Supplemental Information 3 — The histogram of the images obtained from Matlab and FPGA for realistic comparison [file peerj-cs-08-973-s003.zip › 3by3kernel/hist64-128-3.png]

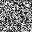

Supplement: Supplemental Information 3 — The histogram of the images obtained from Matlab and FPGA for realistic comparison [file peerj-cs-08-973-s003.zip › 3by3kernel/randomImage32.bmp]

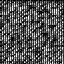

Supplement: Supplemental Information 3 — The histogram of the images obtained from Matlab and FPGA for realistic comparison [file peerj-cs-08-973-s003.zip › 3by3kernel/result_f_0.jpg]

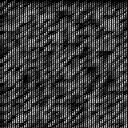

Supplement: Supplemental Information 3 — The histogram of the images obtained from Matlab and FPGA for realistic comparison [file peerj-cs-08-973-s003.zip › 3by3kernel/result_f_1.jpg]

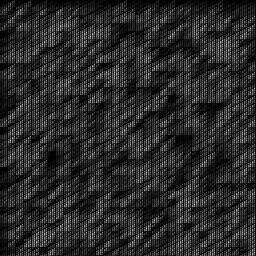

Supplement: Supplemental Information 3 — The histogram of the images obtained from Matlab and FPGA for realistic comparison [file peerj-cs-08-973-s003.zip › 3by3kernel/result_f_2.jpg]

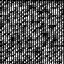

Supplement: Supplemental Information 3 — The histogram of the images obtained from Matlab and FPGA for realistic comparison [file peerj-cs-08-973-s003.zip › 3by3kernel/result_m_0.jpg]

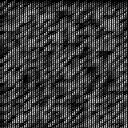

Supplement: Supplemental Information 3 — The histogram of the images obtained from Matlab and FPGA for realistic comparison [file peerj-cs-08-973-s003.zip › 3by3kernel/result_m_1.jpg]

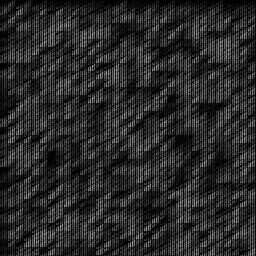

Supplement: Supplemental Information 3 — The histogram of the images obtained from Matlab and FPGA for realistic comparison [file peerj-cs-08-973-s003.zip › 3by3kernel/result_m_2.jpg]

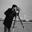

Supplement: Supplemental Information 3 — The histogram of the images obtained from Matlab and FPGA for realistic comparison [file peerj-cs-08-973-s003.zip › 5by5kernel/cameraman32.bmp]

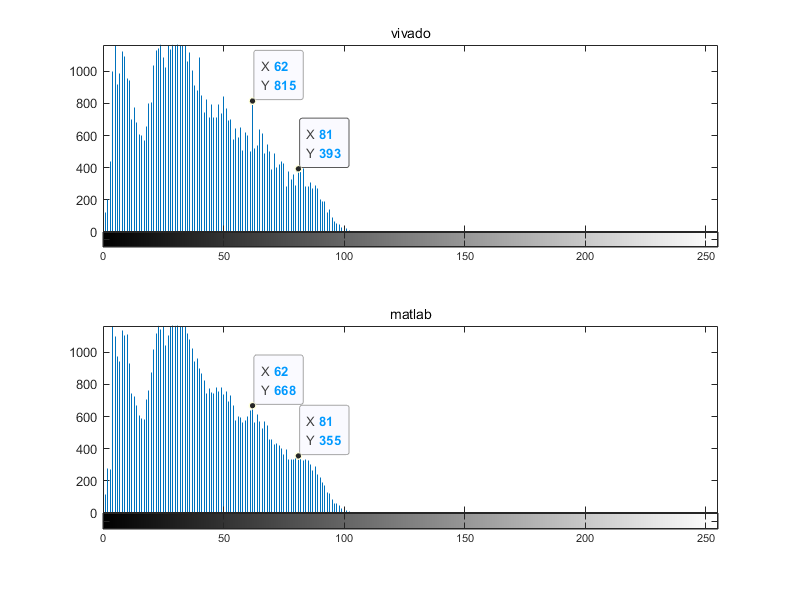

Supplement: Supplemental Information 3 — The histogram of the images obtained from Matlab and FPGA for realistic comparison [file peerj-cs-08-973-s003.zip › 5by5kernel/hist128-256-5.png]

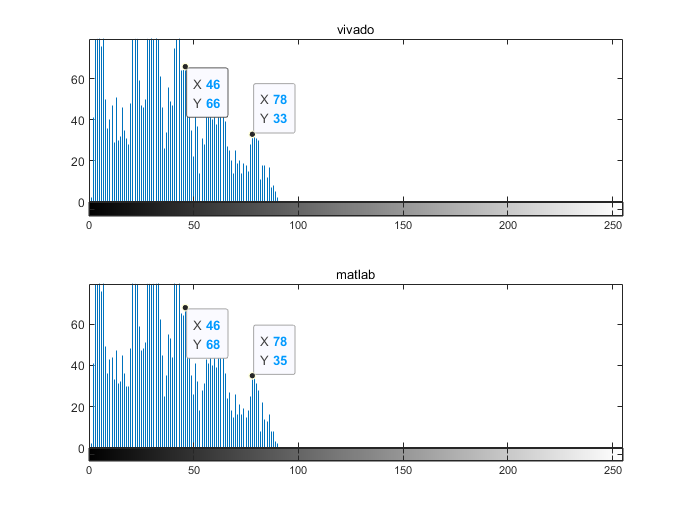

Supplement: Supplemental Information 3 — The histogram of the images obtained from Matlab and FPGA for realistic comparison [file peerj-cs-08-973-s003.zip › 5by5kernel/hist32-64-5.png]

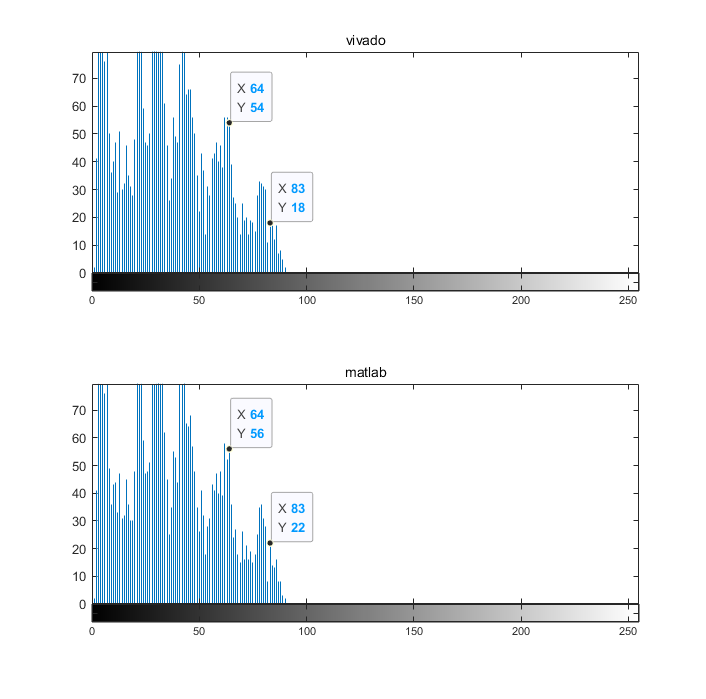

Supplement: Supplemental Information 3 — The histogram of the images obtained from Matlab and FPGA for realistic comparison [file peerj-cs-08-973-s003.zip › 5by5kernel/hist64-128-5.png]

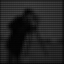

Supplement: Supplemental Information 3 — The histogram of the images obtained from Matlab and FPGA for realistic comparison [file peerj-cs-08-973-s003.zip › 5by5kernel/result_5f_11.png]

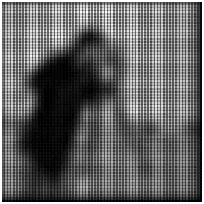

Supplement: Supplemental Information 3 — The histogram of the images obtained from Matlab and FPGA for realistic comparison [file peerj-cs-08-973-s003.zip › 5by5kernel/result_5f_2.png]

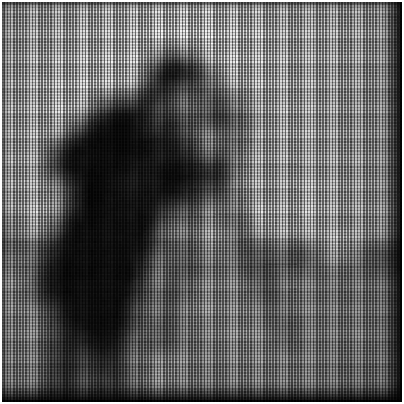

Supplement: Supplemental Information 3 — The histogram of the images obtained from Matlab and FPGA for realistic comparison [file peerj-cs-08-973-s003.zip › 5by5kernel/result_5f_3.png]

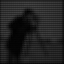

Supplement: Supplemental Information 3 — The histogram of the images obtained from Matlab and FPGA for realistic comparison [file peerj-cs-08-973-s003.zip › 5by5kernel/result_5m_11.png]

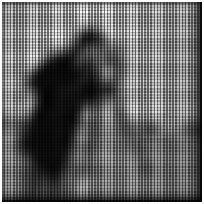

Supplement: Supplemental Information 3 — The histogram of the images obtained from Matlab and FPGA for realistic comparison [file peerj-cs-08-973-s003.zip › 5by5kernel/result_5m_2.png]

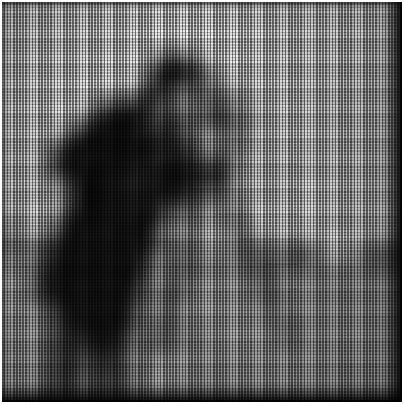

Supplement: Supplemental Information 3 — The histogram of the images obtained from Matlab and FPGA for realistic comparison [file peerj-cs-08-973-s003.zip › 5by5kernel/result_5m_3.png]

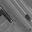

Supplement: Supplemental Information 3 — The histogram of the images obtained from Matlab and FPGA for realistic comparison [file peerj-cs-08-973-s003.zip › 7by7kernel/305_32.jpg]

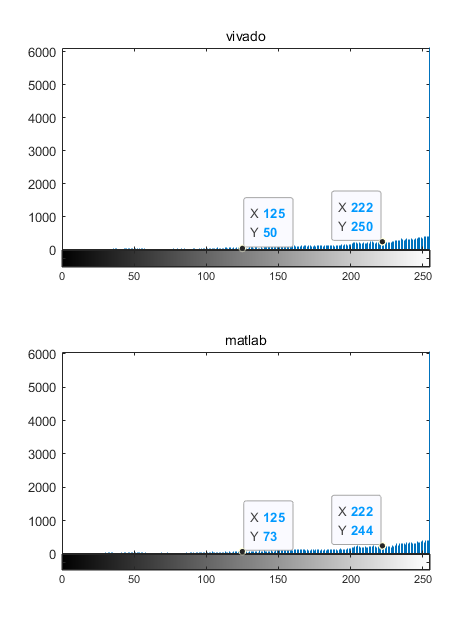

Supplement: Supplemental Information 3 — The histogram of the images obtained from Matlab and FPGA for realistic comparison [file peerj-cs-08-973-s003.zip › 7by7kernel/hist128-256-7.png]

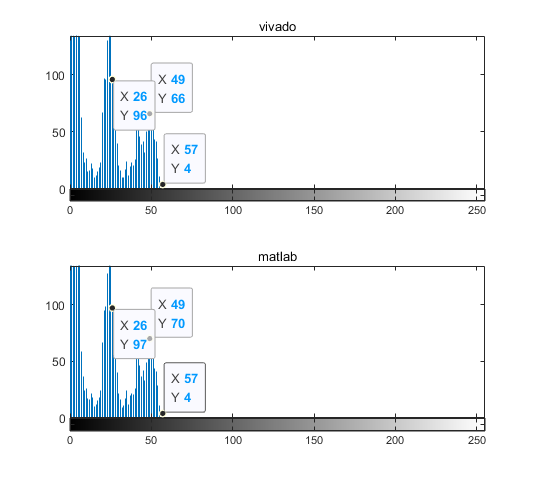

Supplement: Supplemental Information 3 — The histogram of the images obtained from Matlab and FPGA for realistic comparison [file peerj-cs-08-973-s003.zip › 7by7kernel/hist32-64-7.png]

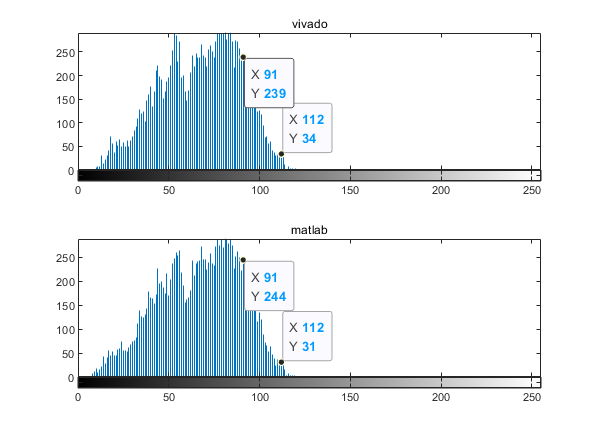

Supplement: Supplemental Information 3 — The histogram of the images obtained from Matlab and FPGA for realistic comparison [file peerj-cs-08-973-s003.zip › 7by7kernel/hist64-128-7.png]

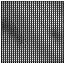

Supplement: Supplemental Information 3 — The histogram of the images obtained from Matlab and FPGA for realistic comparison [file peerj-cs-08-973-s003.zip › 7by7kernel/result_7f_1.jpg]

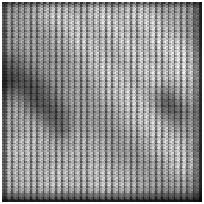

Supplement: Supplemental Information 3 — The histogram of the images obtained from Matlab and FPGA for realistic comparison [file peerj-cs-08-973-s003.zip › 7by7kernel/result_7f_2.jpg]

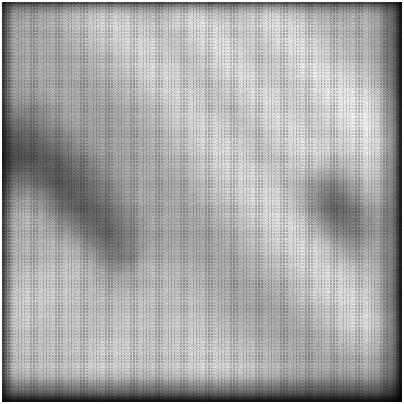

Supplement: Supplemental Information 3 — The histogram of the images obtained from Matlab and FPGA for realistic comparison [file peerj-cs-08-973-s003.zip › 7by7kernel/result_7f_3.jpg]

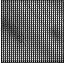

Supplement: Supplemental Information 3 — The histogram of the images obtained from Matlab and FPGA for realistic comparison [file peerj-cs-08-973-s003.zip › 7by7kernel/result_7m_1.jpg]

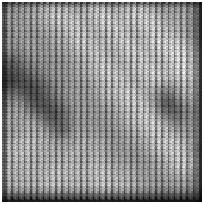

Supplement: Supplemental Information 3 — The histogram of the images obtained from Matlab and FPGA for realistic comparison [file peerj-cs-08-973-s003.zip › 7by7kernel/result_7m_2.jpg]

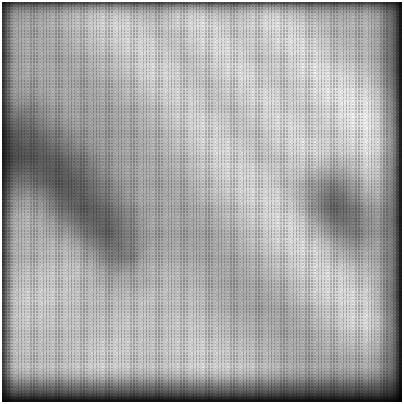

Supplement: Supplemental Information 3 — The histogram of the images obtained from Matlab and FPGA for realistic comparison [file peerj-cs-08-973-s003.zip › 7by7kernel/result_7m_3.jpg]
